# Supplementary material for: Temporal witnesses of non-classicality in a macroscopic biological system
Source: Sci Rep. 2024 Aug 29;14:20094. doi: 10.1038/s41598-024-66159-x (PMC11362292; doi:10.1038/s41598-024-66159-x)
Supplement: Supplementary file 1 — Supplementary Information. [file 41598_2024_66159_MOESM1_ESM.pdf]

Temporal witnesses of non-classicality in a macroscopic biological system - Supplementary Information

(Dated: July 2, 2024)

MARKOVIAN ENVIRONMENT: ANALYTICAL DERIVATION OF EQ.8

In this Appendix, we will analytically derive Eq.8 and the general state for N monomers and R reservoir qubits, going iteration by iteration in the protocol and providing the final state of the emitted photon after the recombination of the exciton on the polymer. The setup is the very same discussed in Section *Markovian vs Non-Markovian Environment*.

First iteration $k = 1$.– We start with the polymer being in the state described by the density matrix in Eq. 3. At this stage, the state of the system is separable, so that we can write:

$$^0\rho_S = {}^0\rho_{S_1} \otimes {}^0\rho_{S_2} \otimes ... \otimes {}^0\rho_{S_N} \tag{S1}$$

being $^0\rho_{S_j}$, $j = 1, ..., N$, the reduced density matrix of the monomer M_j *before* the beginning of the homogenisation process in the $k = 1$ iteration. It is convenient to write the single monomer density matrices using the Bloch vector representation:

$$^0\rho_{S_1} = \frac{1}{2}\left(\mathbf{I} + {}^0\vec{s}_1 \cdot \vec{\sigma}\right) = \frac{1}{2}\left(\mathbf{I} - Z_1\right) \tag{S2}$$

where σ is the vector of Pauli operators and the initial condition for the first monomer of the chain is $^0\vec{s}_1 = -\hat{z}$ since it is excited by the photon, so it is in the state $|1\rangle$. Similarly:

$$^0\rho_{S_j} = \frac{1}{2}\left(\mathbf{I} + {}^0\vec{s}_j \cdot \vec{\sigma}\right) = \frac{1}{2}\left(\mathbf{I} + Z_j\right), \quad j = 2, ..., N \tag{S3}$$

where the initial conditions are $^0\vec{s}_j = +\hat{z}$ because all the remaining monomers are *not* excited by the photon at the beginning. Recall in fact that our photon is initially spatially localised on the first monomer. Moving now to the reservoir qubits, we can adopt the Bloch vector representation for them too:

$$^0\xi_j = \frac{1}{2}\left(\mathbf{I} + {}^0\vec{r}_j \cdot \vec{\sigma}\right) = \frac{1}{2}\left(\mathbf{I}\right), \quad j = 1, ..., R \tag{S4}$$

where $^0\vec{r}_j = 0$ because we initialise all the qubits in the reservoir in the maximally mixed state.

We can now start with the decoherence phase of the $k = 1$ iteration of the protocol, i.e., the first round of homogenisation for the polymer. We start with the first, excited, monomer. It interacts with the first qubit in the reservoir:

$$^1\rho_{S_1}^{(1)} = Tr_{^0\xi_1}\left[\mathrm{PSWAP}_{^0\rho_{S_1}, {}^0\xi_1}\left({}^0\rho_{S_1} \otimes {}^0\xi_1\right)\mathrm{PSWAP}^\dagger_{^0\rho_{S_1}, {}^0\xi_1}\right] = \frac{1}{2}\left(\mathbf{I} + {}^1\vec{s}_1^{(1)} \cdot \vec{\sigma}\right) = \frac{1}{2}\left(\mathbf{I} - \cos^2\eta \; Z_1\right) \tag{S5}$$

where the Bloch vector $^1\vec{s}_1^{(1)}$ evolves as:

$$^1\vec{s}_1^{(1)} = \cos^2\eta \; {}^0\vec{s}_1 + \sin^2\eta \; {}^0\vec{r}_1 - \cos\eta\sin\eta\left({}^0\vec{r}_1 \times {}^0\vec{s}_1\right) = -\cos^2\eta \; \hat{z}. \tag{S6}$$

This means that the decoherence process, at this stage of the protocol, shrinks the length of the monomer's Bloch vector of a factor $\cos^2\eta$ for every qubit in the reservoir. We can easily generalise this process to the Rth interaction with the reservoir, to have:

$$^1\rho_{S_1}^{(R)} = \frac{1}{2}\left(\mathbf{I} + {}^1\vec{s}_1^{(R)} \cdot \vec{\sigma}\right) = \frac{1}{2}\left(\mathbf{I} - \cos^{2R}\eta \; Z_1\right) \tag{S7}$$

where:

$$^1\vec{s}_1^{(R)} = \cos^2\eta \; {}^1\vec{s}_1^{(R-1)} + \sin^2\eta \; {}^0\vec{r}_R - \cos\eta\sin\eta\left({}^0\vec{r}_R \times {}^0\vec{s}_1^{(R-1)}\right) = \cos^2\eta(-\cos^{2(R-1)}\eta)\hat{z} = -\cos^{2R}\eta \; \hat{z}. \tag{S8}$$

This concludes the decoherence phase for the first monomer.

We move now to the second monomer. Since we are in the Markov environment scenario, we can initialise again the reservoir to a collection of R qubits prepared in the maximally mixed state, or equivalently we can say that the

second monomer interacts with a different environment, equally built and prepared. This allows us to use the very same results we got for the decoherence phase of the first monomer, with the only difference that the initial condition for the second monomer M_2 is given by ${}^0\vec{s}_2 = +\hat{z}$ since it is not excited by the photon. We will thus have:

$$ {}^1\rho_{S_2}^{(R)} = \frac{1}{2}\left(\mathbf{I} + {}^1\vec{s}_2^{(R)} \cdot \vec{\sigma}\right) = \frac{1}{2}\left(\mathbf{I} + \cos^{2R}\eta \; Z_2\right) \tag{S9} $$

where:

$$ {}^1\vec{s}_2^{(R)} = \cos^2\eta \; {}^1\vec{s}_2^{(R-1)} + \sin^2\eta \; {}^0\vec{r}_R - \cos\eta\sin\eta\left({}^0\vec{r}_R \times {}^0\vec{s}_2^{(R-1)}\right) = \cos^2\eta(+\cos^{2(R-1)}\eta)\hat{z} = +\cos^{2R}\eta \; \hat{z}. \tag{S10} $$

The same reasoning can be applied to all the remaining monomers in the chain, so that at the end of the decoherence phase for the $k = 1$ iteration of the protocol our monomers will be in the states:

$$ \begin{cases} {}^1\rho_{S_1}^{(R)} = \frac{1}{2}\left(\mathbf{I} - \cos^{2R}\eta \; Z_1\right) \\ {}^1\rho_{S_j}^{(R)} = \frac{1}{2}\left(\mathbf{I} + \cos^{2R}\eta \; Z_j\right) \qquad j = 2, ..., N \end{cases} \tag{S11} $$

and the state of the polymer:

$$ {}^1\rho_S = {}^1\rho_{S_1}^{(R)} \otimes {}^1\rho_{S_2}^{(R)} \otimes ... \otimes {}^1\rho_{S_N}^{(R)}. \tag{S12} $$

The first iteration of the protocol ends with the *transfer phase* involving the monomers M_1 and M_2. This is described by the unitary ($\hbar = 1$ from now on):

$$ U = e^{-iH_{XX}t} \tag{S13} $$

where H_{XX} is the XX Hamiltonian in Eq.5. We find:

$$ {}^1\rho_{S_{1,2}} = U\left({}^1\rho_{S_1}^{(R)} \otimes {}^1\rho_{S_2}^{(R)}\right)U^\dagger \tag{S14} $$

which, at first order in t, gives:

$$ {}^1\rho_{S_{1,2}} = {}^1\rho_{S_1}^{(R)} \otimes {}^1\rho_{S_2}^{(R)} + \frac{1}{4}itJ_1\cos^{2R}\eta\left(\sigma_1^+\sigma_2^- - \sigma_1^-\sigma_2^+\right). \tag{S15} $$

All in all, the state of the polymer after the $k = 1$ iteration of the protocol, which will in turn be the initial state for the $k = 2$ iteration, is:

$$ {}^2\rho_S = {}^1\rho_{S_{1,2}} \otimes {}^1\rho_{S_j}^{(R)\otimes(N-2)} \qquad j = 3, ..., N. \tag{S16} $$

Second iteration $k = 2$.– Since we are dealing with the Markov environment, before beginning with the decoherence phase we must initialise again the reservoir in the state ${}^0\xi$ of Eq.6. The state of the polymer is the one in Eq.S16 instead, where the monomers M_1 and M_2 are entangled. In this case, the homogenisation process will be performed *locally* on both M_1 and M_2. The entangled state ${}^1\rho_{S_{1,2}}$ in Eq.S15 can be divided into a separable and a non-separable part, which we can discuss separately.

The separable part of Eq.S15 will evolve as described in the $k = 1$ iteration of the protocol, with the only difference that now the initial conditions for the two monomers M_1 and M_2 are given by ${}^1\vec{s}_1^{(R)}$ in Eq.S8 and ${}^1\vec{s}_2^{(R)}$ in Eq.S10. After R interactions with the reservoir, we will have;

$$ \begin{cases} {}^2\rho_{S_1}^{(R)} = \frac{1}{2}\left(\mathbf{I} - \cos^{(4R)}\eta Z_1\right) \\ {}^2\rho_{S_2}^{(R)} = \frac{1}{2}\left(\mathbf{I} + \cos^{(4R)}\eta Z_2\right) \end{cases} \tag{S17} $$

The *non*-separable part is the most interesting to explore. We shall call it ${}^1\rho_{S_{1,2}}^{non-sep}$. Let us start with the first monomer being involved in the decoherence phase. Interacting with the first qubit of the reservoir, we find:

$$ {}^1\rho_{S_{1,2}}^{non-sep,(1)} = \frac{1}{2}itJ_1\cos^{2R}\eta\cos^2\eta\left(\sigma_1^+\sigma_2^- - \sigma_1^-\sigma_2^+\right) \tag{S18} $$

so that, after the interaction with the remaining $R-1$ qubits in the reservoir, ${}^1\rho_{S_{1,2}}^{non-sep}$ will be:

$$ {}^1\rho_{S_{1,2}}^{non-sep,(R)} = 2^{(R-2)}itJ_1\cos^{4R}\eta\left(\sigma_1^+\sigma_2^- - \sigma_1^-\sigma_2^+\right). \tag{S19} $$

The same reasoning can be applied when the second monomer M_2 undergoes the decoherence phase. After the interaction with the $R-th$ qubit in the reservoir, the final state of the non-separable part of ${}^1\rho_{S_{1,2}}$ is:

$$ {}^1\rho_{S_{1,2}}^{non-sep,(2R)} = 2^{(2R-2)} itJ_1 \cos^{6R}\eta \left(\sigma_1^+\sigma_2^- - \sigma_1^-\sigma_2^+\right). \tag{S20} $$

All in all, the entangled state in Eq.S15 after the decoherence phase of the $k=2$ iteration becomes (re-scaling the coefficient properly to remove the constant numeric factors):

$$ {}^2\rho_{S_{1,2}}^{(R)} = {}^2\rho_{S_1}^{(R)} \otimes {}^2\rho_{S_2}^{(R)} + itJ_1 \cos^{6R}\eta \left(\sigma_1^+\sigma_2^- - \sigma_1^-\sigma_2^+\right), \tag{S21} $$

while the remaining $N-2$ monomers, not being involved in the quantum state transfer, evolve as described in the previous iteration:

$$ {}^2\rho_{S_j}^{(R)} = \frac{1}{2}\left(\mathbf{I} + \cos^{4R}\eta Z_j\right) \qquad j = 3, ..., N. \tag{S22} $$

We move now to the transfer phase of the $k=2$ iteration, involving the monomers M_2 and M_3. Applying again the unitary in Eq.S13 to first order in t, we find:

$$ \begin{aligned} {}^2\rho_{S_{1,2,3}} = U\left({}^2\rho_{S_{1,2}}^{(R)} \otimes {}^2\rho_{S_3}^{(R)}\right)U^\dagger = \Big[{}^2\rho_{S_1}^{(R)} \otimes {}^2\rho_{S_2}^{(R)} + itJ_1\cos^{6R}\eta\left(\sigma_1^+\sigma_2^- - \sigma_1^-\sigma_2^+\right) - t^2 B_2\left(\sigma_1^+\sigma_2^- + \sigma_1^-\sigma_2^+\right)\Big] \otimes {}^2\rho_{S_3}^{(R)} \\ - t^2 J_1 J_2 \cos^{6R}\eta\left(Z_2 + \cos^{4R}\eta\right)\left(\sigma_1^+\sigma_3^- + \sigma_1^-\sigma_3^+\right). \end{aligned} \tag{S23} $$

We can now summarise the outcome of the $k=2$ iteration of the protocol, which in turn is the initial state of the iteration $k=3$. The state of the polymer will be:

$$ {}^3\rho_S = {}^2\rho_{S_{1,2,3}} \otimes {}^2\rho_{S_j}^{(R)\otimes(N-3)} \qquad j = 4, ..., N. \tag{S24} $$

$N-th$ iteration $k=N$ and recombination.– Once we have understood how our decoherence map works for the decoherence phase and what mechanisms are involved in the transfer phase, we can easily generalise the protocol to the very last step: the exciton arrives at the last but one monomer in the chain and, after the last decoherence phase, is transmitted to the monomer M_N; it is at this stage that the exciton will finally recombine on the polymer, emitting again a single photon. The final state of the photon will thus be:

$$ \begin{aligned} {}^N\rho_p = \Big[{}^N\rho_{p_1} \otimes {}^N\rho_{p_2} + itJ_1\cos^{[2+4(N-2)]R}\eta\left(\alpha_1^+\alpha_2^- - \alpha_1^-\alpha_2^+\right) - t^2 B_2 \cos^{4(N-3)R}\eta\left(\alpha_1^+\alpha_2^- + \alpha_1^-\alpha_2^+\right)\Big] \, {}^N\rho_{p_3} \otimes ... \otimes {}^N\rho_{p_N} \\ - t^2 J_1 J_2 \cos^{[2+4(N-2)]R}\eta\left(\cos^{(N-3)2R}\eta Z_2 + \cos^{4R}\eta\right)\left[\left(\alpha_1^+\alpha_3^- + \alpha_1^-\alpha_3^+\right) + itB_3\left(\alpha_1^+\alpha_3^- - \alpha_1^-\alpha_3^+\right)\right] {}^N\rho_{p_4} \otimes ... \otimes {}^N\rho_{p_N} \\ + ... + (i)^{2N-3} t^{N-2} J_1 J_2 ... J_{N-1} \cos^{[2+4(N-2)]R}\eta\left(\cos^{(N-3)2R}\eta Z_2 + \cos^{4R}\eta\right) ... \left(Z_N + \cos^{2(N-1)R}\eta\right) \cdot \\ \cdot\left[\left(\alpha_1^+\alpha_N^- + (-1)^{2N-3}\alpha_1^-\alpha_N^+\right) + itB_N\left(\alpha_1^+\alpha_N^- + (-1)^{2N-2}\alpha_1^-\alpha_N^+\right)\right]. \end{aligned} \tag{S25} $$

where ${}^N\rho_{p_j}$ is the density matrix of the photon localised over the monomer M_j at the $k=N$ iteration of the protocol.

NON-MARKOVIAN ENVIRONMENT: ANALYTICAL DERIVATION OF EQ.9

Here we will detail the procedure leading to Eq.9 and provide some useful generalisation to compute the needed coefficients for larger systems. The setup is the very same as discussed in Section *Markovian vs Non-Markovian Environment.*

First iteration $k=1$.– We start again with the polymer being in the state described by the density matrix in Eq. 3:

$$ {}^0\rho_S = {}^0\rho_{S_1} \otimes {}^0\rho_{S_2} \otimes {}^0\rho_{S_3} \tag{S26} $$

being ${}^0\rho_{S_j}$, $j = 1, ..., 3$, the reduced density matrix of the monomer M_j *before* the beginning of the homogenisation process in the $k=1$ iteration. We write the single monomer density matrices using the Bloch vector representation:

$$ {}^0\rho_{S_1} = \frac{1}{2}\left(\mathbf{I} + {}^0\vec{s}_1 \cdot \vec{\sigma}\right) = \frac{1}{2}\left(\mathbf{I} - Z_1\right) \tag{S27} $$

where σ is the vector of Pauli operators and the initial condition for the first monomer of the chain is ${}^0\vec{s}_1 = -\hat{z}$ since it is excited by the photon, so it is in the state $|1\rangle$. Similarly:

$$ {}^0\rho_{S_j} = \frac{1}{2}\left(\mathbf{I} + {}^0\vec{s}_j \cdot \vec{\sigma}\right) = \frac{1}{2}\left(\mathbf{I} + Z_j\right), \quad j = 2, 3 \tag{S28} $$

where the initial conditions are ${}^0\vec{s}_j = +\hat{z}$ because all the remaining monomers are *not* excited by the photon at the beginning. Recall in fact that our photon is initially spatially localised on the first monomer. Moving now to the reservoir qubits, we can adopt for them too the Bloch vector representation:

$$ {}^0\xi_j = \frac{1}{2}\left(\mathbf{I} + {}^0\vec{r}_j \cdot \vec{\sigma}\right) = \frac{1}{2}\left(\mathbf{I}\right), \quad j = 1, ..., 3 \tag{S29} $$

where ${}^0\vec{r}_j = 0$ because we initialise all the qubits in the reservoir in the maximally mixed state.

We can now start with the *decoherence phase* of the $k = 1$ iteration of the protocol, i.e., the first round of homogenisation for the polymer. We start with the first, excited, monomer. It interacts with the first qubit in the reservoir:

$$ {}^1\rho^{(1)}_{S_1} = Tr_{{}^0\xi_1}\left[\text{PSWAP}_{{}^0\rho_{S_1},{}^0\xi_1}\left({}^0\rho_{S_1} \otimes {}^0\xi_1\right)\text{PSWAP}^\dagger_{{}^0\rho_{S_1},{}^0\xi_1}\right] = \frac{1}{2}\left(\mathbf{I} + {}^1\vec{s}^{(1)}_1 \cdot \vec{\sigma}\right) = \frac{1}{2}\left(\mathbf{I} - \cos^2\eta \; Z_1\right) \tag{S30} $$

where the Bloch vector ${}^1\vec{s}^{(1)}_1$ evolves as:

$$ {}^1\vec{s}^{(1)}_1 = \cos^2\eta \; {}^0\vec{s}_1 + \sin^2\eta \; {}^0\vec{r}_1 - \cos\eta \sin\eta \left({}^0\vec{r}_1 \times {}^0\vec{s}_1\right) = -\cos^2\eta \; \hat{z}. \tag{S31} $$

As before, the decoherence process on the first monomer has the effect of shrinking the length of its Bloch vector of a factor $\cos^2\eta$ for every qubit in the reservoir.

Here comes the difference with the previous scenario: since now the environment we are dealing with is a Non-Markovian one, we have to *store* in the reservoir qubits the information about the interaction with the first monomer of the chain. Thus, we have also to compute the states of the reservoir qubits *after* the interaction with M_1:

$$ {}^1\xi'_1 = Tr_{{}^0\rho_{S_1}}\left[\text{PSWAP}_{{}^0\rho_{S_1},{}^0\xi_1}\left({}^0\rho_{S_1} \otimes {}^0\xi_1\right)\text{PSWAP}^\dagger_{{}^0\rho_{S_1},{}^0\xi_1}\right] = \frac{1}{2}\left(\mathbf{I} + {}^1\vec{r}^{(1)}_1 \cdot \vec{\sigma}\right) = \frac{1}{2}\left(\mathbf{I} - \sin^2\eta \; Z^{res}_1\right) \tag{S32} $$

where the Bloch vector ${}^1\vec{r}^{(1)}_1$ evolves as:

$$ {}^1\vec{r}^{(1)}_1 = \sin^2\eta \; {}^0\vec{s}_1 + \cos^2\eta \; {}^0\vec{r}_1 + \cos\eta \sin\eta \left({}^0\vec{r}_1 \times {}^0\vec{s}_1\right) = -\sin^2\eta \; \hat{z}. \tag{S33} $$

Since the reservoir qubits are all, at this stage, initialised in the maximally mixed state, we can easily generalise this process to the 3rd interaction of the monomer M_1 with the reservoir, to have:

$$ {}^1\rho^{(3)}_{S_1} = \frac{1}{2}\left(\mathbf{I} + {}^1\vec{s}^{(3)}_1 \cdot \vec{\sigma}\right) = \frac{1}{2}\left(\mathbf{I} - \cos^6\eta \; Z_1\right) \tag{S34} $$

where:

$$ {}^1\vec{s}^{(3)}_1 = \cos^2\eta \; {}^1\vec{s}^{(2)}_1 + \sin^2\eta \; {}^0\vec{r}_3 - \cos\eta \sin\eta \left({}^0\vec{r}_3 \times {}^0\vec{s}^{(2)}_1\right) = \cos^2\eta(-\cos^4\eta)\hat{z} = -\cos^6\eta \; \hat{z}, \tag{S35} $$

while the second and third reservoir qubit will be left in the states:

$$ {}^1\xi'_2 = \frac{1}{2}\left(\mathbf{I} + {}^1\vec{r}^{(1)}_2 \cdot \vec{\sigma}\right) = \frac{1}{2}\left(\mathbf{I} - \sin^2\eta \cos^2\eta \; Z^{res}_2\right) \tag{S36} $$

and

$$ {}^1\xi'_3 = \frac{1}{2}\left(\mathbf{I} + {}^1\vec{r}^{(1)}_3 \cdot \vec{\sigma}\right) = \frac{1}{2}\left(\mathbf{I} - \sin^2\eta \cos^4\eta \; Z^{res}_3\right), \tag{S37} $$

where:

$$ {}^1\vec{r}^{(1)}_2 = \sin^2\eta \; {}^1\vec{s}^{(1)}_1 + \cos^2\eta \; {}^0\vec{r}_2 + \cos\eta \sin\eta \left({}^0\vec{r}_2 \times {}^1\vec{s}^{(1)}_1\right) = -\sin^2\eta \cos^2\eta \; \hat{z} \tag{S38} $$

and

$$ {}^{1}\vec{r}_3^{(1)} = \sin^2\eta\ {}^{1}\vec{s}_1^{(2)} + \cos^2\eta\ {}^{0}\vec{r}_3 + \cos\eta\sin\eta\left({}^{0}\vec{r}_3 \times {}^{1}\vec{s}_1^{(2)}\right) = -\sin^2\eta\cos^4\eta\ \hat{z}. \tag{S39}$$

Now is the turn of the second monomer M_2 to undergo the decoherence phase. The qubits in the reservoir are now in the states ${}^{1}\xi_j^{'}$, $j = 1, 2, 3$. The process is the same described before, giving at the end of the interaction of M_2 with the third qubit in the reservoir:

$$ {}^{1}\rho_{S_2}^{(3)} = \frac{1}{2}\left[\mathbf{I} + \cos^4\eta\left(\cos^2\eta - 3\sin^4\eta\right) Z_2\right] \tag{S40}$$

and

$$\begin{cases} {}^{1}\xi_1^{''} = \frac{1}{2}\left[\mathbf{I} + \sin^2\eta\left(1-\cos^2\eta\right)\ Z_1^{res}\right] \\ {}^{1}\xi_2^{''} = \frac{1}{2}\left[\mathbf{I} + \sin^2\eta\left(\cos^2\eta - \cos^4\eta - \sin^4\eta\right)\ Z_2^{res}\right] \\ {}^{1}\xi_3^{''} = \frac{1}{2}\left[\mathbf{I} + \sin^2\eta\cos^2\eta\left(\cos^2\eta - \cos^4\eta - 2\sin^4\eta\right)\ Z_3^{res}\right]. \end{cases} \tag{S41}$$

To conclude the decoherence phase for the $k = 1$ iteration, we move now to the monomer M_3 considering the reservoir qubits in ${}^{1}\xi_j^{''}$, $j = 1, 2, 3$:

$$ {}^{1}\rho_{S_3}^{(3)} = \frac{1}{2}\left\{\left[\mathbf{I} + \left[3\sin^4\eta\cos^2\eta\left(\cos^2\eta - \cos^4\eta - \sin^4\eta\right) + \cos^6\eta\right] Z_3\right\} \tag{S42}$$

and

$$\begin{cases} {}^{1}\xi_1^{'''} = \frac{1}{2}\left\{\mathbf{I} + \left[\sin^2\eta\cos^2\eta\left(1-\cos^2\eta\right) + \sin^2\eta\right] Z_1^{res}\right\} \\ {}^{1}\xi_2^{'''} = \frac{1}{2}\left[\mathbf{I} + \frac{1}{32}\sin^2\eta(3\cos 2\eta + 6\cos 4\eta - 3\cos 6\eta + 26) Z_2^{res}\right] \\ {}^{1}\xi_3^{'''} = \frac{1}{2}\left[\mathbf{I} + \frac{1}{64}\sin^2\eta(58\cos 2\eta - 8\cos 4\eta + 6\cos 6\eta - 3\cos 8\eta + 11) Z_3^{res}\right]. \end{cases} \tag{S43}$$

To conclude the first iteration of the protocol we move now to the *transfer phase* involving the monomers M_1 and M_2. The unitary describing this process is the one we introduced in Eq.S13, so that:

$$ {}^{1}\rho_{S_{1,2}} = U\left({}^{1}\rho_{S_1}^{(3)} \otimes {}^{1}\rho_{S_2}^{(3)}\right) U^{\dagger} \tag{S44}$$

which gives, at first order in t:

$$ {}^{1}\rho_{S_{1,2}} = {}^{1}\rho_{S_1}^{(3)} \otimes {}^{1}\rho_{S_2}^{(3)} + itJ_1\cos^4\eta\left(\cos^2\eta - \frac{3}{2}\sin^4\eta\right)\left(\sigma_1^+\sigma_2^- - \sigma_1^-\sigma_2^+\right). \tag{S45}$$

This concludes the first iteration of our protocol, leaving the polymer in the state:

$$ {}^{2}\rho_S = {}^{1}\rho_{S_{1,2}} \otimes {}^{1}\rho_{S_3}^{(3)} \tag{S46}$$

and the reservoir qubits in the states in Eq.S43.

Second iteration $k = 2$ and recombination.– We move now to the second and final iteration of the protocol before the recombination of the exciton and, differently from the Markov scenario, the initial conditions for the reservoir will be given by the states in Eq.S43, as we want the environment to keep *memory* of the previous steps of the process. This brings in some differences in the way in which separable and non-separable parts of Eq.S45 evolve, which we will attention now.

Let us start with the first monomer M_1, which interacts with the first qubit in the reservoir with the usual PSWAP:

$$ {}^{2}\rho_{S_{1,2}}^{(1)} = Tr_{{}^{2}\xi_1^{'''}}\left[\text{PSWAP}\left({}^{1}\rho_{S_{1,2}} \otimes {}^{1}\xi_1^{'''}\right)\text{PSWAP}^{\dagger}\right] \tag{S47}$$

$$ = Tr_{{}^{2}\xi_1^{''}}\left[\text{PSWAP}\left({}^{1}\rho_{S_1}^{(3)} \otimes {}^{1}\rho_{S_2}^{(3)} \otimes {}^{1}\xi_1^{'''}\right)\text{PSWAP}^{\dagger}\right] \tag{S48}$$

$$ + itJ_1\cos^4\eta\left(\cos^2\eta - \frac{3}{2}\sin^4\eta\right) Tr_{{}^{2}\xi_1^{''}}\left[\text{PSWAP}\left(\left(\sigma_1^+\sigma_2^- - \sigma_1^-\sigma_2^+\right) \otimes {}^{1}\xi_1^{'''}\right)\text{PSWAP}^{\dagger}\right] \tag{S49}$$

The separable part in Eq.S48 evolves as explained before, with the only difference that now the initial conditions will be given by ${}^{1}\vec{s}_1^{(3)}$ in Eq.S35 and ${}^{1}\vec{r}_1^{(3)} = \sin^2\eta\cos^2\eta(1-\cos^2\eta)+\sin^2\eta$, so to have:

$$ {}^{2}\rho_{S_1}^{(1)} \otimes {}^{2}\rho_{S_2}^{(0)} = \frac{1}{2}\left\{\mathbf{I} + {}^{2}s_1^{(1)}(\eta) Z_1\right\} \otimes {}^{2}\rho_{S_2}^{(0)}, \tag{S50}$$

where:

$$ {}^{2}s_1^{(1)}(\eta) = -\left[\cos^8\eta + \frac{1}{8}\sin^4\eta\,(\cos 4\eta - 9)\right] \tag{S51}$$

is the $\hat{z}$ component of the Bloch vector ${}^{2}\vec{s}_1$ after the interaction with the first reservoir qubit.

Moving now to the non separable part in Eq.S49, that we will call here ${}^{1}\rho_{S_{1,2}}^{non-sep}$, we have:

$$ {}^{1}\rho_{S_{1,2}}^{non-sep,(1)} = itJ_1\cos^4\eta\left(\cos^2\eta - \frac{3}{2}\sin^4\eta\right)\left[\cos^2\eta\left(\sigma_1^+\sigma_2^- - \sigma_1^-\sigma_2^+\right)\right. \tag{S52}$$

$$ \left. + i\sin^3\eta\cos\eta\left(\cos^2\eta - \cos^4\eta + 1\right)\left(\sigma_1^+\sigma_2^- + \sigma_1^-\sigma_2^+\right)\right], \tag{S53}$$

that we can conveniently write as:

$$ {}^{1}\rho_{S_{1,2}}^{non-sep,(1)} = itJ_1\left[F^{(1,0)}(\eta)\left(\sigma_1^+\sigma_2^- - \sigma_1^-\sigma_2^+\right)\right. \tag{S54}$$

$$ \left. + G^{(1,0)}(\eta)\left(\sigma_1^+\sigma_2^- + \sigma_1^-\sigma_2^+\right)\right] \tag{S55}$$

where:

$$ \begin{cases} F^{(1,0)}(\eta) = \cos^6\eta\left(\cos^2\eta - \frac{3}{2}\sin^4\eta\right) \\ G^{(1,0)}(\eta) = i\cos^4\eta\sin^5\eta\left(\cos^2\eta - \frac{3}{2}\sin^4\eta\right)\left(\cos^2\eta - \cos^4\eta + 1\right). \end{cases} \tag{S56}$$

The superscript (l,m) indicates the stage of the interactions of the two entangled monomers with the reservoir: the first label refers to M_1, the second to M_2. Comparing this result with Eq.S18, we notice that a new hopping term appears, in Eq.S55, which shows a π-phase difference with the one in Eq.S54: it comes from the coherence of the reservoir qubit due to its non-markovianity. Interestingly, the phase factor would have been different depending on the orientation of the reservoir qubit's Bloch vector $\vec{r}_1$ on the Bloch sphere: in our model, it is a π-phase because ${}^{1}\vec{r}_1^{(3)}$ is parallel to the $\hat{z}$-axis of the sphere.
Before moving on, we have to update the state of the first reservoir qubit, because of the non-markovianity of the environment we are dealing with:

$$ {}^{2}\xi_1' = \frac{1}{2}\left[\mathbf{I} + {}^{2}r_1'(\eta) Z_1^{res}\right] \tag{S57}$$

where:

$$ {}^{2}r_1'(\eta) = \cos^2\eta\sin^4\eta\,(2+\cos 2\eta). \tag{S58}$$

Here ${}^{2}r_1'(\eta)$ is the $\hat{z}$ component of the Bloch vector ${}^{2}\vec{r}_1$ after the interaction with the monomer M_1.

We can now proceed with the second monomer M_2 and its interaction with the first qubit in the reservoir. Splitting again separable and non-separable parts as before, we find:

$$ {}^{2}\rho_{S_1}^{(1)} \otimes {}^{2}\rho_{S_2}^{(1)} = {}^{2}\rho_{S_1}^{(1)} \otimes \frac{1}{2}\left[\mathbf{I} + {}^{2}s_2^{(1)} Z_2\right], \tag{S59}$$

where

$$ {}^{2}s_2^{(1)} = \cos^2\eta\left(\cos^{10}\eta + \sin^4\eta + \cos^2\eta\sin^4\eta - 5\cos^4\eta\sin^4\eta\right), \tag{S60}$$

and:

$$ {}^{1}\rho_{S_{1,2}}^{non-sep,(2)} = itJ_1\left[F^{(1,1)}(\eta)\left(\sigma_1^+\sigma_2^- - \sigma_1^-\sigma_2^+\right)\right. \tag{S61}$$

$$ \left. + G^{(1,1)}(\eta)\left(\sigma_1^+\sigma_2^- + \sigma_1^-\sigma_2^+\right)\right] \tag{S62}$$

being:

$$\begin{cases} F^{(1,1)}(\eta) = \cos^2\eta F^{(1,0)}(\eta) + i\sin\eta\cos\eta G^{(1,0)}(\eta)^2 r_1^{'}(\eta) \\ G^{(1,1)}(\eta) = \cos^2\eta G^{(1,0)}(\eta) - i\sin\eta\cos\eta F^{(1,0)}(\eta)^2 r_1^{'}(\eta). \end{cases} \tag{S63}$$

Before moving on with the third monomer M_3, we have to extract the new state of the first reservoir qubit:

$$^2\xi_1^{''} = \frac{1}{2}\left[\mathbf{I} + {}^2r_1^{''}(\eta) Z_1^{res}\right] \tag{S64}$$

where:

$$^2r_1^{''}(\eta) = \cos^4\eta\sin^2\eta\left(\cos^2\eta + \cos^6\eta - 5\sin^4\eta + 1\right). \tag{S65}$$

We can thus conclude the interactions with the first reservoir qubit considering the monomer M_3. At the $k = 2$ iteration of the protocol, it has not been involved in the transfer phase yet, meaning that it is not entangled with the monomers M_1 and M_2. We simply have to write then:

$$^2\rho_{S_3}^{(1)} = Tr_{^2\xi_1^{''}}\left[\text{PSWAP}\left({}^1\rho_{S_3}^{(3)} \otimes {}^2\xi_1^{''}\right)\text{PSWAP}^\dagger\right] = \frac{1}{2}\left\{\mathbf{I} + {}^2s_3^{(1)} Z_3\right\}, \tag{S66}$$

with:

$$^2s_3^{(1)} = \left[\cos^8\eta + \sin^4\eta\cos^4\eta\left(4\cos^2\eta - 3\cos^4\eta + \cos^6\eta - 8\sin^4\eta + 1\right)\right], \tag{S67}$$

and:

$$^2\xi_1^{'''} = Tr_{^1\rho_{S_3}^{(3)}}\left[\text{PSWAP}\left({}^1\rho_{S_3}^{(3)} \otimes {}^2\xi_1^{''}\right)\text{PSWAP}^\dagger\right] = \frac{1}{2}\left[\mathbf{I} + {}^2r_1^{'''} Z_1^{res}\right], \tag{S68}$$

where:

$$^2r_1^{'''} = -\frac{1}{64}\cos^2\eta\sin^2\eta\,(25 - 332\cos 2\eta + 68\cos 4\eta - 20\cos 6\eta + 3\cos 8\eta) \tag{S69}$$

We have now to move to the second and third reservoir qubits in our Non-Markov environment going through the very same steps outlined above. The state of the polymer at end of the decoherence phase of the $k = 2$ iteration of the protocol in the Non-Markov scenario will then be:

$$^2\rho_S^{(3)} = {}^2\rho_{S_{1,2}}^{(3)} \otimes {}^2\rho_{S_3}^{(3)} \tag{S70}$$

where:

$$^2\rho_{S_{1,2}}^{(3)} = {}^2\rho_{S_1}^{(3)} \otimes {}^2\rho_{S_2}^{(3)} + itJ_1\left[F(\eta)\left(\sigma_1^+\sigma_2^- - \sigma_1^-\sigma_2^+\right) + G(\eta)\left(\sigma_1^+\sigma_2^- + \sigma_1^-\sigma_2^+\right)\right] \tag{S71}$$

with:

$$^2\rho_{S_1}^{(3)} = \frac{1}{2}\left[\mathbf{I} + {}^2s_1^{(3)} Z_1\right], \tag{S72}$$

$$^2s_1^{(3)} = \frac{1}{2048}\left(1302 - 128\cos 2\eta + 655\cos 4\eta + 128\cos 6\eta + 90\cos 8\eta + \cos 12\eta\right); \tag{S73}$$

$$^2\rho_{S_2}^{(3)} = \frac{1}{2}\left[\mathbf{I} + {}^2s_2^{(3)} Z_2\right], \tag{S74}$$

$$^2s_2^{(3)} = \left[\cos^{16}\eta + \sin^{12}\eta - \frac{\cos^2\eta\sin^4\eta}{128}\left(-147 - 18\cos 2\eta + 12\cos 4\eta + 17\cos 6\eta + 7\cos 8\eta + \cos 10\eta\right)\right], \tag{S75}$$

and:

$$F(\eta) \coloneqq F^{(3,3)}(\eta) = \cos^2\eta F^{(3,2)}(\eta) + i\sin\eta\cos\eta G^{(3,2)}(\eta)^2 r_3^{'}$$
$$= -\frac{1}{2.48\cdot 10^{27}}\left[\cos^{14}\eta\left(1.84\cdot 10^{27}\cos 2\eta - 2.62\cdot 10^{27}\cos 4\eta + 9.33\cdot 10^{26}\cos 6\eta - 8.97\cdot 10^{25}\cos 8\eta - 1.34\cdot 10^{26}\cos 10\eta\right.\right.$$
$$+1.63\cdot 10^{26}\cos 12\eta - 1.22\cdot 10^{26}\cos 14\eta + 6.98\cdot 10^{25}\cos 16\eta - 3.50\cdot 10^{25}\cos 18\eta + 1.54\cdot 10^{25}\cos 20\eta - 6.13\cdot 10^{24}\cos 22\eta$$
$$+2.23\cdot 10^{24}\cos 24\eta - 7.00\cdot 10^{23}\cos 26\eta + 1.72\cdot 10^{23}\cos 28\eta - 2.23\cdot 10^{22}\cos 30\eta - 7.94\cdot 10^{21}\cos 32\eta + 8.59\cdot 10^{21}\cos 34\eta$$
$$-4.83\cdot 10^{21}\cos 36\eta + 2.14\cdot 10^{21}\cos 38\eta - 8.00\cdot 10^{20}\cos 40\eta + 2.53\cdot 10^{20}\cos 42\eta - 6.56\cdot 10^{19}\cos 44\eta + 1.16\cdot 10^{19}\cos 46\eta$$
$$+3.15\cdot 10^{17}\cos 48\eta - 1.42\cdot 10^{18}\cos 50\eta + 7.92\cdot 10^{17}\cos 52\eta - 3.05\cdot 10^{17}\cos 54\eta + 8.99\cdot 10^{16}\cos 56\eta - 1.94\cdot 10^{16}\cos 58\eta$$
$$+1.99\cdot 10^{15}\cos 60\eta + 6.90\cdot 10^{14}\cos 62\eta - 5.02\cdot 10^{14}\cos 64\eta + 1.85\cdot 10^{14}\cos 66\eta - 4.80\cdot 10^{13}\cos 68\eta + 7.93\cdot 10^{12}\cos 70\eta$$
$$+2.69\cdot 10^{10}\cos 72\eta - 5.41\cdot 10^{11}\cos 74\eta + 1.90\cdot 10^{11}\cos 76\eta - 3.09\cdot 10^{10}\cos 78\eta + 5.51\cdot 10^{8}\cos 80\eta + 7.30\cdot 10^{8}\cos 82\eta$$
$$\left.\left.-1.18\cdot 10^{8}\cos 84\eta + 2.79\cdot 10^{6}\cos 86\eta + 9.61\cdot 10^{5}\cos 88\eta - 7.31\cdot 10^{4}\cos 90\eta - 1.78\cdot 10^{3}\cos 92\eta + 243\cos 94\eta - 2.49\cdot 10^{27}\right)\right] \tag{S76}$$

$$G(\eta) \coloneqq G^{(3,3)}(\eta) = \cos^2\eta G^{(3,2)}(\eta) - i\sin\eta\cos\eta F^{(3,2)}(\eta)^2 r_3^{'}$$
$$= \frac{i\sin^3\eta\cos^{13}\eta}{1.51\cdot 10^{23}}\left[-5.43\cdot 10^{22}\cos 2\eta + 1.01\cdot 10^{23}\cos 4\eta + 8.20\cdot 10^{21}\cos 6\eta + 2.12\cdot 10^{22}\cos 8\eta + 5.32\cdot 10^{21}\cos 10\eta\right.$$
$$-3.36\cdot 10^{21}\cos 12\eta + 2.26\cdot 10^{21}\cos 14\eta - 1.53\cdot 10^{21}\cos 16\eta + 7.24\cdot 10^{20}\cos 18\eta - 1.96\cdot 10^{20}\cos 20\eta + 3.24\cdot 10^{19}\cos 22\eta$$
$$+1.64\cdot 10^{19}\cos 24\eta - 1.83\cdot 10^{19}\cos 26\eta + 1.09\cdot 10^{19}\cos 28\eta - 5.12\cdot 10^{18}\cos 30\eta + 1.88\cdot 10^{18}\cos 32\eta - 5.17\cdot 10^{17}\cos 34\eta$$
$$+9.20\cdot 10^{16}\cos 36\eta + 2.65\cdot 10^{15}\cos 38\eta - 1.24\cdot 10^{16}\cos 40\eta + 7.07\cdot 10^{15}\cos 42\eta - 2.61\cdot 10^{15}\cos 44\eta + 6.79\cdot 10^{14}\cos 46\eta$$
$$-1.02\cdot 10^{14}\cos 48\eta - 6.60\cdot 10^{12}\cos 50\eta + 1.12\cdot 10^{13}\cos 52\eta - 4.94\cdot 10^{12}\cos 54\eta + 1.50\cdot 10^{12}\cos 56\eta - 2.95\cdot 10^{11}\cos 58\eta$$
$$+7.72\cdot 10^{9}\cos 60\eta + 1.83\cdot 10^{10}\cos 62\eta - 6.35\cdot 10^{9}\cos 64\eta + 8.40\cdot 10^{8}\cos 66\eta + 3.30\cdot 10^{7}\cos 68\eta - 2.53\cdot 10^{7}\cos 70\eta$$
$$\left.+2.84\cdot 10^{6}\cos 72\eta - 3.28\cdot 10^{4}\cos 74\eta - 1.41\cdot 10^{4}\cos 76\eta + 729\cos 78\eta + 7.17\cdot 10^{22}\right] \tag{S77}$$

while:

$${}^2\rho_{S_3}^{(3)} = \frac{1}{2}\left[\mathbf{I} + s(\eta)Z_3\right], \tag{S78}$$

$s(\eta)$ being:

$$s(\eta) = {}^2s_3^{(3)} = \cos^{12}\eta + \sin^{12}\eta + \sin^4\eta\left(3\cos^8\eta + 6\cos^{10}\eta - 3\cos^{12}\eta + 4\cos^{14}\eta\right) + \sin^8\eta\left(6\cos^4\eta + 7\cos^6\eta - 27\cos^8\eta\right)$$
$$+\sin^{12}\eta\left(-\cos^2\eta - 2\cos^4\eta - 4\cos^6\eta\right). \tag{S79}$$

These complex coefficients are shown as a function of the decoherence parameter η in Fig1.

Once the decoherence phase ends, the transfer phase of the $k = 2$ iteration of the protocol can take place. We thus apply again the unitary in Eq.S13 to the state ${}^2\rho_S^{(3)}$ in Eq.S70 to obtain:

$${}^3\rho_S = {}^3\rho_{S_1}\otimes{}^3\rho_{S_2}\otimes{}^3\rho_{S_3} + itJ_1\left[F(\eta) + 2itB_2G(\eta)\right]\left(\sigma_1^+\sigma_2^- - \sigma_1^-\sigma_2^+\right)\otimes{}^3\rho_{S_3}$$
$$+ itJ_1\left[G(\eta) + 2itB_2F(\eta)\right]\left(\sigma_1^+\sigma_2^- + \sigma_1^-\sigma_2^+\right)\otimes{}^3\rho_{S_3}$$
$$-\frac{1}{2}t^2J_1J_2\left(Z_2 + s(\eta)\right)\left[F(\eta)\left(\sigma_1^+\sigma_3^- + \sigma_1^-\sigma_3^+\right) + G(\eta)\left(\sigma_1^+\sigma_3^- - \sigma_1^-\sigma_3^+\right)\right] \tag{S80}$$

so that, after the recombination of the exciton, the emitted photon is in the state in Eq.9.

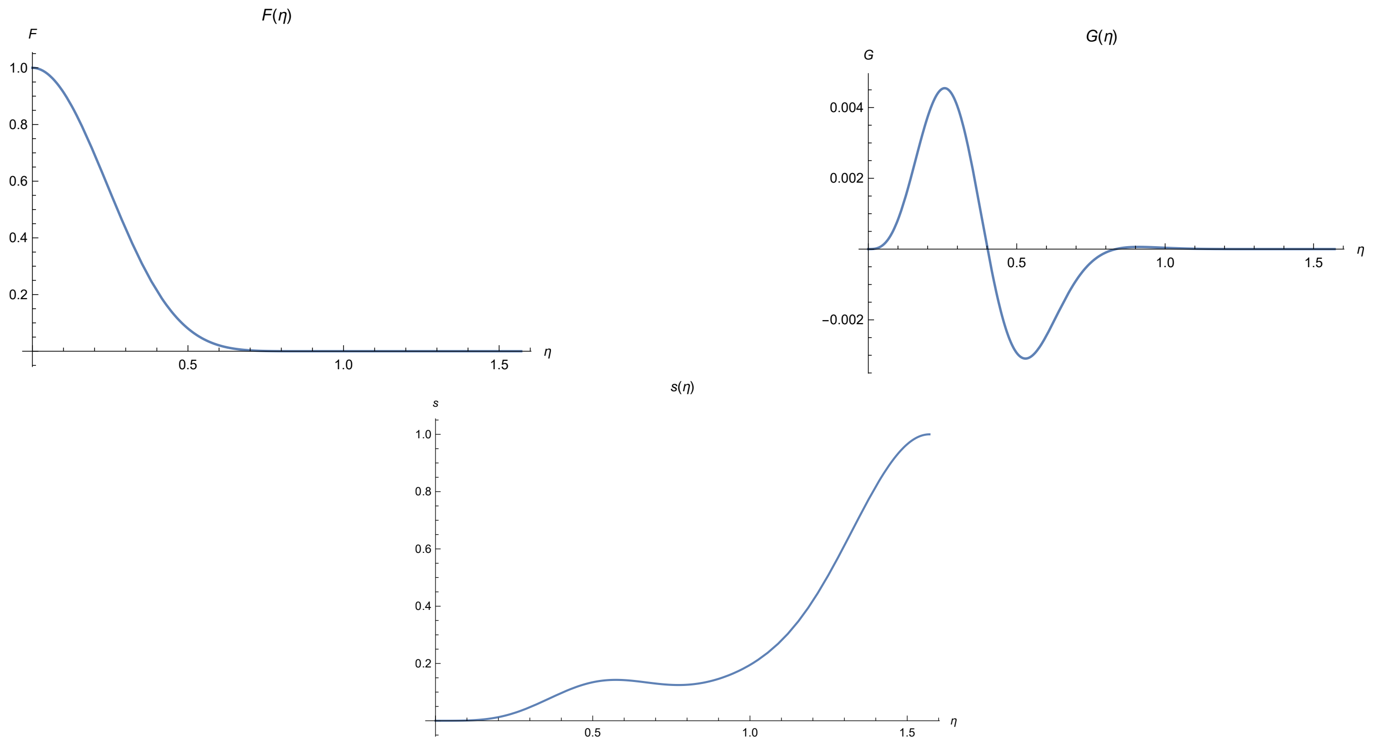

Figure 1. Coefficient $F(\eta)$, $G(\eta)$ and $s(\eta)$ at the end of $k = 2$ iteration.

COMPARISON BETWEEN $F(\eta)$, $G(\eta)$ AND THE MARKOVIAN EQUIVALENT FOR THE EXCITON TRANSFER *VIA THE CHAIN*

In this Appendix, we include the tables detailing the values of the coefficients of the terms describing the coherent delocalisation of the exciton *via the chain* for different values of the decoherence parameter η. The goal of this Appendix is to support the claims that in the weak coupling regime (1) $F(\eta)$ and its Markovian counterpart have the same value *at every step* of the protocol and (2) that $G(\eta)$ is small enough to be negligible.

$\eta = 0.01$	Markov	$F(\eta)$	$G(\eta)$
$M_1 \leftrightarrow Q_1$	0.9996	0.9996	$i9.9 \cdot 10^{-7}$
$M_1 \leftrightarrow Q_2$	0.9995	0.9995	$i9.9 \cdot 10^{-7}$
$M_1 \leftrightarrow Q_3$	0.9994	0.9994	$i1.9 \cdot 10^{-6}$
$M_2 \leftrightarrow Q_1$	0.9993	0.9993	$i3.9 \cdot 10^{-10}$
$M_2 \leftrightarrow Q_2$	0.9992	0.9992	$i9.9 \cdot 10^{-7}$
$M_2 \leftrightarrow Q_3$	0.9991	0.9991	$-i9.9 \cdot 10^{-7}$

$\eta = 0.1$	Markov	$F(\eta)$	$G(\eta)$
$M_1 \leftrightarrow Q_1$	0.961	0.961	$i9.7 \cdot 10^{-4}$
$M_1 \leftrightarrow Q_2$	0.951	0.951	$i9.3 \cdot 10^{-4}$
$M_1 \leftrightarrow Q_3$	0.942	0.942	$i1.3 \cdot 10^{-3}$
$M_2 \leftrightarrow Q_1$	0.932	0.932	$i3.7 \cdot 10^{-5}$
$M_2 \leftrightarrow Q_2$	0.923	0.923	$i9.4 \cdot 10^{-4}$
$M_2 \leftrightarrow Q_3$	0.914	0.914	$-i8.3 \cdot 10^{-4}$

$\eta = 1.0$	Markov	$F(\eta)$	$G(\eta)$
$M_1 \leftrightarrow Q_1$	$7.263 \cdot 10^{-3}$	$-1.173 \cdot 10^{-2}$	$-i1.52 \cdot 10^{-2}$
$M_1 \leftrightarrow Q_2$	$2.120 \cdot 10^{-3}$	$-1.736 \cdot 10^{-3}$	$-i3.2 \cdot 10^{-3}$
$M_1 \leftrightarrow Q_3$	$6.189 \cdot 10^{-4}$	$7.826 \cdot 10^{-5}$	$-i9.3 \cdot 10^{-4}$
$M_2 \leftrightarrow Q_1$	$1.807 \cdot 10^{-4}$	$2.117 \cdot 10^{-4}$	$-i3.1 \cdot 10^{-4}$
$M_2 \leftrightarrow Q_2$	$5.275 \cdot 10^{-5}$	$7.715 \cdot 10^{-5}$	$-i8.8 \cdot 10^{-5}$
$M_2 \leftrightarrow Q_3$	$1.540 \cdot 10^{-5}$	$3.501 \cdot 10^{-5}$	$-i3.6 \cdot 10^{-5}$

Table I. Coefficients hopping terms via chain with $\eta = 0.01$, $\eta = 0.1$ and $\eta = 1.0$ at every step of the decoherence phase of $k = 2$ iteration.

Table I shows the coefficients for $\eta = 0.01$, $\eta = 0.1$ and $\eta = 1.0$. One can see that in the weak coupling regime, claims (1) and (2) are satisfied, but as soon as we increase the decoherence parameter the coefficients $F(\eta)$ and their

Markovian counterpart become dissimilar. In contrast, $G(\eta)$ cannot be neglected anymore as it has the same order of magnitude as $F(\eta)$. We leave the reader to the main text for an interpretation of these results.
